# Radically reframing studies on neurobiology and socioeconomic circumstances: A call for social justice-oriented neuroscience

**DOI:** 10.3389/fnint.2022.958545

**Published:** 2022-09-02

**Authors:** E. Kate Webb, Carlos Cardenas-Iniguez, Robyn Douglas

**Affiliations:** ^1^Department of Psychology, University of Wisconsin–Milwaukee, Milwaukee, WI, United States; ^2^Department of Psychiatry, Harvard Medical School, Boston, MA, United States; ^3^Division of Depression and Anxiety, McLean Hospital, Belmont, MA, United States; ^4^Department of Population and Public Health Sciences, University of Southern California, Los Angeles, CA, United States; ^5^Department of Psychological and Behavioral Sciences, Texas A&M University, College Station, TX, United States

**Keywords:** socioeconomic position (SEP), neighborhood disadvantage, neurobiology of stress, social justice, structural racism

## Abstract

Socioeconomic circumstances are associated with symptoms and diagnostic status of nearly all mental health conditions. Given these robust relationships, neuroscientists have attempted to elucidate how socioeconomic-based adversity “gets under the skin.” Historically, this work emphasized individual proxies of socioeconomic position (e.g., income, education), ignoring the effects of broader socioeconomic contexts (e.g., neighborhood socioeconomic disadvantage) which may uniquely contribute to chronic stress. This omission represented a disconnect between neuroscience and other allied fields that have recognized health is undeniably linked to interactions between systems of power and individual characteristics. More recently, neuroscience work has considered how sociopolitical context affects brain structure and function; however, the products of this exciting line of research have lacked critical sociological and historical perspectives. While empirical evidence on this topic is burgeoning, the cultural, ethical, societal, and legal implications of this work have been elusive. Although the mechanisms by which socioeconomic circumstances impact brain structure and function may be similar across people, not everyone is exposed to these factors at similar rates. Individuals from ethnoracially minoritized groups are disproportionally exposed to neighborhood disadvantage. Thus, socioeconomic inequities examined in neuroscience research are undergirding with other forms of oppression, namely structural racism. We utilize a holistic, interdisciplinary approach to interpret findings from neuroscience research and interweave relevant theories from the fields of public health, social sciences, and Black feminist thought. In this perspective piece, we discuss the complex relationship that continues to exist between academic institutions and underserved surrounding communities, acknowledging the areas in which neuroscience research has historically harmed and/or excluded structurally disadvantaged communities. We conclude by envisioning how this work can be used; not just to inform policymakers, but also to engage and partner with communities and shape the future direction of human neuroscience research.

## Introduction

*“Radical simply means ‘grasping things at the root’.”*

*–Angela Davis*

Much of human research has centered on how adversity, including lower individual socioeconomic position (SEP), becomes biologically embedded (Turner and Lloyd, 1995; McEwen, 2012a; McLaughlin and Sheridan, 2016). With evidence from physiology, genomics, and neuroimaging, our knowledge regarding the impact of socioeconomic circumstances on mental health has progressed remarkably (Hackman and Farah, 2009; Gianaros and Hackman, 2013; Brito and Noble, 2014; Johnson et al., 2016; Farah, 2017, 2018). Despite considerable empirical evidence demonstrating the biological burden of socioeconomic factors, attempts to deliver evidence-based interventions to address these types of adversity have been laborious and with few victories (Wainberg et al., 2017; Campion et al., 2022). We propose this impasse is because the majority of human neuroscience work does not systematically include these factors in study designs or situate findings within existing social inequities, including structural racism [definitions of terms used throughout the article are provided in Table 1 (Gee and Ford, 2011; Sewell, 2015; Riley, 2018; Yearby, 2020)].

**TABLE 1:** Terminology and definitions.

Term	Definition
Inequities	Differences (e.g., between ethnoracial groups, between socioeconomic positions, etc.) which are unjust, unfair, and avoidable (Bailey et al., 2017; Krieger, 2021). *Inequalities*, a closely-related concept, refer to the measured difference in a particular outcome (Krieger, 2001).
Chronic stress	Repeated exposures to myriad multi-level risk factors (e.g., work stress, trauma, environmental toxins, community violence, police brutality, etc.) and unstable access to necessary resources (e.g., education, food, transportation, etc.) (McEwen and Gianaros, 2010; Kim et al., 2018).
Structural racism	“The macro-level systems, social forces, institutions, ideologies, and processes that interact with one another to generate and reinforce inequalities among racial and ethnic groups” (Gee and Ford, 2011).
Law	The mechanisms of legal systems, including the political processes, policies, and legal practices such as enforcement (Yearby, 2020).
Critical race theory	A framework used to analyze the historical and contemporary forms of structural racism (Crenshaw, 2010).
Positionality	How a person’s sociopolitical identity (e.g., gender identity, sexual identity, race, ethnicity, socioeconomic position, religion, etc.) and lived experiences shape their position in society. Ultimately, this position influences how a person interacts with and perceives the world (Roberts S. O. et al., 2020).
Intersectionality	Rooted in Black feminist pedagogy, a framework used to analyze “relations between systems of oppression which construct our multiple identities and our social locations in hierarchies of power and privilege” (Crenshaw, 1991; Carastathis, 2014).

Historically, mental health research braved the matter of social inequities. However, in the 1980’s, a shift towards biological perspectives caused the focus to diminish (Muntaner et al., 2000; Bernard, 2006; Dean, 2018). More explicitly, while research on physical health has increasingly built upon social determinants of health and disease (Krieger, 2011, 1994), the dominant narrative in mental health research embraced biological models of disease. This shift decreased the number of studies investigating how structural drivers of social determinants (e.g., sociopolitical context, legal frameworks, and policies) impacted individuals (Muntaner et al., 2000; Krieger, 2001; Crear-Perry et al., 2021).

In a similar vein, the association between neurobiology and neighborhood socioeconomic factors (e.g., neighborhood disadvantage) has received even less attention than associations with individual-level variables (e.g., income or education Farah, 2017, 2018). This may reflect study design limitations; there is simplicity in collecting individual-level measures directly from the participant, and the benefit of evading the expenses associated with larger sample sizes, which are often required to observe significant effects of neighborhood factors. Another explanation of this trend is that neuroscience research has been implicitly biased towards using a “Freedom” model of health, which suggests people are solely responsible for their health and related behaviors i.e., individual-oriented theories of disease causation (Dougherty, 1993; Muntaner et al., 2000; Krieger, 2001, 2011). This line of thinking perpetuates harmful stereotypes of genetic inferiority and pathologizes those living amongst poor socioeconomic conditions (Farah, 2018), as it attributes health disparities along sociodemographic categories to the individual or essential characteristics of members of the marginalized group.

Though many issues arise when defaulting to the Freedom model, perhaps most insidious is that it complements the “deserving poor” argument or “boot-strap” ideology, which alleges people are in specific socioeconomic positions because of individual differences in ambition or talent. To be clear, this stance is not reflected in data. In fact, upward mobility rates in the United States have continued to decline over the past 10 years. Variables capturing the effects of structural racism, such as race and place (e.g., region, neighborhood) remain the strongest predictors of mobility (Connor and Storper, 2020). Thus, the “Freedom” model—and those akin to it—disregards the longstanding inequities in opportunity in the United States and, when applied (consciously or not) to neuroscience research, exonerates the oppressive structures which maintain inequities.

Broad mechanistic questions about socioeconomic circumstances can be challenging to capture because the measures are generally considered macro factors, instead of proximate mechanisms which interact directly with an individual’s neurobiology. “For this reason,” as it refers to the reason why socioeconomic circumstances can be challenging to captur. However, various models have highlighted the myriad ways our social systems can interact with the brain, as the brain works in part as a social organ, consistently informed by our interactions with our environment (Lende and Downey, 2012; Berman et al., 2019). Further, dimensions of socioeconomic circumstances, such as social and material conditions, are related to other, more proximal factors, which have causal roles in mental health risk. On the environmental side, these proximal factors can include prenatal and postnatal nutritional deficiencies and SEP-linked exposures to environmental toxins. They also include the interaction of crucial non-physical socioeconomic factors such as parental education.

We focused on studies of neighborhood disadvantage and neurobiology in this perspective because research in this area inherently emphasizes *place* and *context* rather than the *individual*. This work marks a recent and fervent shift toward recognizing that the broader sociopolitical context affects how individuals interpret stimuli and navigate within social groups. This further highlights the need for the field to firmly declare that societal inequities exist and are relevant to the understanding of brain structure and function. Few neuroscientists (if any) would endorse the contrary, but by excluding these variables and disregarding societal influences, the resulting scientific products lack this context. By including variables at multiple levels that better capture the forces and dynamics related to SEP in human neuroscience experiments, researchers acknowledge that some of the variability in individual differences—whether in biological functioning, behavioral task performance, or clinical symptoms—is attributable to the sociopolitical stratification in society (Gianaros and Hackman, 2013).

Studies on the relationship between socioeconomic factors and neurobiology are at the forefront and intersection of public health, neuroscience, and sociology, and in this perspective paper, we leverage knowledge across these disciplines. After briefly reviewing theories linking socioeconomic factors to mental health, we highlight evidence that neighborhood disadvantage is associated with neurobiology. This work would be strengthened by positioning research questions and findings within sociological and historical context. Although we center neighborhood disadvantage, the issues presented in this article are shared with studies on individual SEP and are relevant to all human neuroscience research. Individual SEP and neighborhood disadvantage may impact biological systems through different mechanisms. However, socioeconomic variables at multiple levels share structural racism as an upstream determinant.

We call for future studies to name structural racism, define neighborhood disadvantage as an institutionalized form of racial inequity, and interpret how the effects of racism are captured in methods and manifest in results (Sewell, 2015, 2016; Riley, 2018). Finally, we describe areas and steps for improvement, including acknowledging historical and current inequities, reporting relevant data, and funding research that prioritizes the needs and participation of historically excluded communities. These recommendations are based in the belief that neuroscience could more critically address mental health disparities if an anti-racist radical framework—which considers the root causes of inequities—was applied.

### Theories linking socioeconomic factors to health

Researchers have developed various socioecological theories to better understand how environmental exposure can uniquely interact with genotypes and phenotypes to differentially impact human development and mental health (Ellis et al., 2011). For example, Social Causation Theory posits that poorer socioeconomic circumstances increase an individual’s risk for mental health conditions, including post-traumatic stress disorder (PTSD), depressive disorder, and generalized anxiety disorders (Hollingshead and Redlich, 1958). This increased risk is partially due to greater environmental resource scarcity and higher environmental stress, which may affect neurocognitive development in childhood and adolescence (Farah, 2018; Ferschmann et al., 2022). For an individual, alterations in neurocognitive development may represent biological risk for mental health conditions. Over time, these effects may reduce socioeconomic achievement in adulthood, creating intergenerational patterns of socioeconomic-related stress for oppressed communities (Hackman et al., 2010).

The timing and accumulation of factors associated with poorer socioeconomic circumstances across the lifecourse are also identified as a crucial element in frameworks focusing on the embodiment and embedding of social, structural, and environmental factors and their relevance to biological development and functioning. Though a detailed discussion of “lifecourse exposome” studies is outside the scope of this article (see Evans and Kim, 2012; Kelly-Irving and Delpierre, 2021; Vineis and Barouki, 2022), these approaches highlight the importance of dynamic upstream structural, sociopolitical, and temporal factors in the study of biological and psychological functioning.

Another set of theories focuses on individual differences in genomic variations and how these may be related to an individual’s susceptibility to eventual mental health symptoms. Differential Susceptibility Theory advances the claim that individuals can inherently differ in their susceptibility to stressors, and that individuals’ environments may interact with genetic variations and behavioral outcomes “for better or worse” (Belsky and Pluess, 2009; Ellis et al., 2011). Through this theory, researchers have focused on identifying the moderating influence of environmental exposures on developmental and life outcomes. For example, previous work in this area has focused on psychological markers such as negative emotionality as potentially significant individual susceptibility factors (Ellis et al., 2011). In Differential Susceptibility Theory, both positive (e.g., supportive parenting) and negative (e.g., neighborhood disadvantage) environmental conditions are theorized to influence an individual’s susceptibility to mental health outcomes.

A contrasting model is the Diathesis-Stress Model, which suggests that individuals have a baseline level of predisposing factors (i.e., diathesis) for any given mental health condition. The point at which individuals develop symptoms depends on the interaction between the risk factors and the degree of stress. One form of diathesis is biological and includes neurophysiological dysregulation. When repeated instances of stress occur, this can cause biological changes that result in more sensitivity to stress in the future, meaning that less stress becomes necessary to activate the requisite processes that may facilitate mental health symptoms (Post, 1992; Ingram and Luxton, 2005). Notably, the Diathesis-Stress Model is considered a deficit-only model, focusing on susceptibility to negative environments.

Together, the reviewed theories highlight the importance of considering mechanisms and factors at various levels in studying mental health outcomes and neurobiology. Though these theories did not originally consider how structural racism explained differences in environmental conditions, new applications of these theories identify racism as a determinant. In order to conduct research on the impact of socioeconomic factors on neurobiology properly and equitably, it is crucial to include structural, social, and historical context, and how this may contribute to differential susceptibility and vulnerability and their impact on health (Diderichsen et al., 2019).

### Neural correlates of neighborhood socioeconomic disadvantage

Neighborhood disadvantage measures [e.g., poverty rate, composite measures such as the area deprivation index or social vulnerability index, concentrated disadvantage, etc.; (Sampson et al., 1997; Coulton et al., 2002; Singh, 2003; Flanagan et al., 2011; Kind et al., 2014; Kind and Buckingham, 2018)], established with a geographical ID and through a process of geo-coding (Fan et al., 2021), predict mental health symptoms, even above individual socioeconomic measures. Greater neighborhood disadvantage is associated with higher stress levels (Steptoe and Feldman, 2001; Aneshensel, 2009; Hackman et al., 2012; Barrington et al., 2014; Chattarji et al., 2015; Snedker and Herting, 2016) and symptoms of depression (Blair et al., 2014), anxiety (Casciano and Massey, 2012; Vine et al., 2012), and PTSD (Gapen et al., 2011; Hall Brown and Mellman, 2014; Douglas et al., 2021).

Biological correlates of neighborhood disadvantage span various biological systems. Researchers have examined the effects across different measures of stress responding, such as cortisol reactivity (Karb et al., 2012; Barrington et al., 2014; Finegood et al., 2017; Zilioli et al., 2017), stress-accelerated aging (Olden et al., 2015; Lei et al., 2018, 2019; Lawrence et al., 2020), and immune system regulation (Kepper et al., 2016; Neergheen et al., 2019; Roberts L. et al., 2020). In nearly all proposed mechanistic models, neighborhood disadvantage is conceptualized as chronic stress and therefore hypothesized to influence mental health *via* stress-responding pathways (e.g., persistent hypothalamic-pituitary-adrenal axis activation; Hackman and Farah, 2009; McEwen and Gianaros, 2010; McEwen, 2012b; Gianaros and Hackman, 2013; Farah, 2017).

**FIGURE 1:**
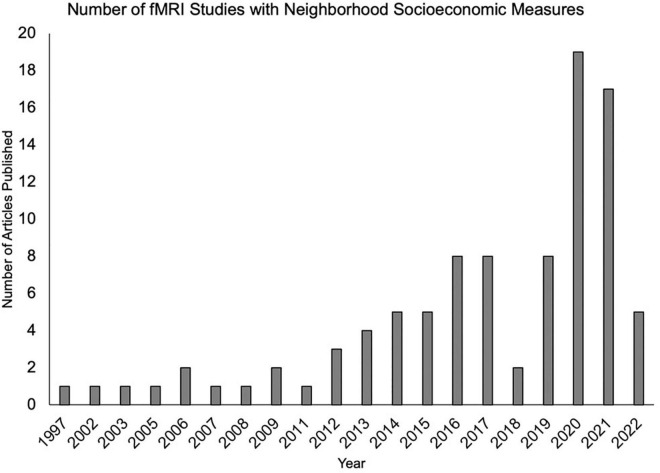
An increasing number of functional magnetic resonance imaging studies are examining neighborhood-level socioeconomic factors. Articles were identified by the authors using a PubMed search which included functional magnetic resonance imaging (fMRI) and at least one neighborhood term (neighborhood disadvantage, neighborhood deprivation, neighborhood poverty, concentrated disadvantage, and concentrated poverty).

The impact of neighborhood disadvantage on neurobiology continues to grow as an exciting line of research (Figure 1). Thanks to large-scale studies such as the Adolescent Brain Cognitive Development (ABCD) study, a number of findings have illustrated the impact of neighborhood disadvantage on brain development, structure, and function (Mullins et al., 2020; Taylor et al., 2020; Vargas et al., 2020; Hackman et al., 2021; Rakesh et al., 2021). Notably, the majority of previous work does not name factors and dynamics related to structural racism. Although a comprehensive and systematic review was outside the scope of this article, we highlight key findings suggesting neighborhood disadvantage is associated with widespread alterations in brain structure and function across the lifespan.

Perhaps most well-documented is a significant association between greater neighborhood disadvantage and decreased prefrontal thickness and smaller hippocampal volumes (Brito and Noble, 2014; Whittle et al., 2017; Wrigglesworth et al., 2019; Hunt et al., 2020; Taylor et al., 2020; Vargas et al., 2020; Webb et al., 2021). Several studies have also found neighborhood disadvantage is associated with lower total surface area and subcortical volume (Hunt et al., 2020; Hackman et al., 2021). In identifying the neurobiological mechanisms linking neighborhood disadvantage to mental health, these structural changes are compelling targets; thinner prefrontal cortex and smaller hippocampus are associated with PTSD and depression (Karl et al., 2006; MacQueen and Frodl, 2011).

Even after accounting for individual SEP, neighborhood disadvantage has been linked to delayed structural and functional neurodevelopmental trajectories (e.g., Ramphal et al., 2020; Tooley et al., 2020; Gard et al., 2021; Rakesh et al., 2021). Rakesh et al. (2021) teased apart the distinct and shared effects of neighborhood disadvantage and household SEP, demonstrating interactive effects between the two different measures on resting-state networks, and further highlighting individual SEP does not fully account for neighborhood effects. Task-based neuroimaging indicates neighborhood disadvantage helps explain individual differences in affective and cognitive domains (Gard et al., 2018; Tomlinson et al., 2020; Huggins et al., 2022; Tomas et al., 2022). For example, Tomlinson and colleagues demonstrated neighborhood disadvantage was related to neural and behavioral correlates of response inhibition (i.e., cognitive domain). In adolescents, neighborhood disadvantage was associated with greater amygdala reactivity to ambiguous neutral faces (Gard et al., 2018) and, in adults, neighborhood disadvantage was related to diminished amygdala threat-related activity (Harnett et al., 2017; i.e., affective domains). These findings point to environmentally driven changes, suggesting living in disadvantaged neighborhoods elicits activity in various neural processes which may place additional demands on cognitive resources. These resources may be bidirectionally exacerbated by the structural changes evoked by living in a chronically stressful environment. Together, these modifications to brain structure and function may create susceptibility to mental health disorders.

Although more empirical work is needed, this theory helps explain why individuals residing in more disadvantaged neighborhoods report more mental health symptoms (Gapen et al., 2011; Casciano and Massey, 2012; Vine et al., 2012; Blair et al., 2014; Hall Brown and Mellman, 2014; Douglas et al., 2021). Although the mechanisms by which neighborhood disadvantage impacts brain structure and function may be fundamentally the same across people, not everyone is exposed to this factor at the same rates. Individuals from ethnoracially minoritized groups are disproportionally exposed to neighborhood disadvantage.

In all the aforementioned work, researchers were faced with methodological decisions concerning the intersections between race, ethnicity, SEP, and neighborhood disadvantage. Despite strong theoretical support that ethnoracial inequities and socioeconomic inequities are related but not equivalent (Williams, 1999), the ability to statistically tease apart these effects is challenging. Others (e.g., Nuru-Jeter et al., 2018) have provided recommendations on how to statistically approach measures of ethnoracial and socioeconomic inequities. Given that upstream sociopolitical and structural factors interact with processes at all levels of analysis, it is critical to acknowledge the overlapping patterns of ethnoracial and socioeconomic inequities in studies of neurobiology and related factors, both in their operationalization and conceptualization, to ensure a science that is reproducible, rigorous, and responsible^1^ (Williams and Mohammed, 2013; Nuru-Jeter et al., 2018; Williams, 2018).

### Naming structural racism as a root cause

Socioeconomic inequities influence health independent of race and ethnicity, however, both individual and neighborhood socioeconomic indicators are ethnoracialized (i.e., stratified by race and ethnicity; Williams, 1999; Williams and Mohammed, 2013; Nuru-Jeter et al., 2018; Williams et al., 2019). In this way, the socioeconomic inequities discussed in studies on neighborhood disadvantage and neurobiology are undergirding and intersecting with other forms of oppression, particularly racism (Sewell, 2015). In fact, *all* of the canonically defined social determinants of health (e.g., economic stability, education access, and quality, etc.) can take form and hold power through structural racism (Sewell, 2015, 2016; Nuru-Jeter et al., 2018; Riley, 2018; Yearby, 2020). Certain exposures, such as neighborhood disadvantage, exist as a racialized risk factor because of structural racism (Riley, 2018). Recent empirical evidence underscores the racialization of neighborhoods: Black Americans in middle SEPs are still more likely to live in disadvantaged neighborhoods compared to white Americans in *lower* SEPs (Turner and Greene, 2021).

Further, for racially minoritized communities, such as Black, Indigenous, Latinx, Asian, and Pacific Islanders, acute stressors coupled with historical stressors and trauma (e.g., discrimination) have been linked to long-term adverse health outcomes (Williams and Mohammed, 2013). Chronically elevated cortisol levels and a dysregulated hypothalamic– pituitary–adrenal (HPA) axis have been found to mediate the effects of racial discrimination on allostatic load and disease for communities of color (Berger and Sarnyai, 2015). Neuroimaging studies on the effects of discrimination and social exclusion have suggested greater activity in areas associated with threat processing and vigilance [e.g., anterior cingulate cortex, amygdala, insula (Berger and Sarnyai, 2015; Clark et al., 2018; Han et al., 2020; Fani et al., 2021; Webb et al., 2022)]. Together, these studies suggest compounded stress effects for members of historically minoritized groups, above and beyond those expected from experiencing neighborhood disadvantage.

There have been resounding calls in public health and allied fields for structural racism to be named as the root cause of ethnoracial health disparities and related racialized socioeconomic inequities (Yosso, 2005; Ford and Airhihenbuwa, 2010a,b; Gee and Ford, 2011; Bailey et al., 2017; Hardeman et al., 2018; Yearby, 2020). Still the majority of human neuroscience research has been reluctant to confront structural racism; infrequently naming structural racism in introductions or discussions. To echo a question raised by Sewell (2016): why not “spell out the connections between health disparities and institutional (in)actions rooted in racism?” The addition of historical and sociological perspectives and the explicit naming of structural racism do not hinder or diminish neuroscience; rather, these perspectives complement, advance, and aptly challenge and hold accountable the current state of the research.

### Situating studies within historical and contemporary context

Differential exposure to neighborhood disadvantage is maintained by historical and current ethnoracial residential segregation. Historic redlining is perhaps the most well-known practice contributing to residential segregation (McClure et al., 2019). **Laws** from the 1930’s until 1968 (when redline mapping was made illegal), allowed the government-led Homeowners’ Loan Corporation to create maps for lending institutions (Massey and Denton, 1993; Hillier, 2003; Sewell, 2015; Connolly et al., 2018; McClure et al., 2019). These maps were used to prevent people of color from residing in specific neighborhoods by limiting bank credit and altering real-estate practices (Massey and Denton, 1993). The resulting changes across the entire homebuying process ultimately forced people to buy houses in less “desirable” (redlined) neighborhoods (Massey and Denton, 1993). In addition, these policies and practices resulted in expansive divestment in redlined neighborhoods and disproportionate investment in predominately white neighborhoods.

Redlining may have historic roots, but the legacy in redlined neighborhoods manifests in the lasting neighborhood disadvantage and ultimately in the residents’ mental and physical health (Massey and Denton, 1993; Sewell, 2015; Williams et al., 2019; Park and Quercia, 2020). For instance, recent research suggests Black and Latinx communities in disadvantaged neighborhoods have an increased likelihood of being exposed to air pollution and toxins, the largest environmental health risk factor in the United States, which can have potentially deleterious effects on physical and mental health (Tessum et al., 2019). Studies show this disproportionate burden of pollution exposure is partially caused by the overconsumption of goods and services from white populations, producing toxins that are disproportionality inhaled by Black and Latinx communities (Tessum et al., 2019).

Current housing law and practices are also culpable, people of color are still disproportionately denied fair mortgage loans (Hanifa, 2021) and Black and Latinx communities continue to be under-valued and under-funded (Park and Quercia, 2020). Withholding certain types of investment (e.g., under-funding of schools) while also misallocating funds to non-community approved budgets (e.g., policing) maintains neighborhood disadvantage. Historic and current racist policies and practices force(d) people of color, particularly Black Americans, to disproportionally reside in neighborhood’s experiencing socioeconomic disadvantage. Thus, neighborhood advantage is a protective factor that can be—and has been—bestowed upon white people by law. Even the terms “neighborhood advantage” or “neighborhood disadvantage” fundamentally aligns with language used—in theories of Black feminism and **intersectionality**—to discuss structural racism; white individuals unfairly benefit from these structural advantages and ethnoracially minoritized individuals are harmed.

## Recommendations for radicalizing human neuroscience

In our work as neuroscientists, we must recognize that people live within environmental contexts shaped by sociopolitical stratification. When we study neighborhood disadvantage, we are studying an exposure that is relevant to mental health because of its connection to structural racism (Sewell, 2015; Riley, 2018). In essence, this perspective is a call for the radicalization of human neuroscience work—a necessary paradigm shift that grasps at the roots of the issue rather than dodging them. By remaining silent (i.e., not acknowledging structural racism) in our work, we fail to hold the institutions protecting structural racism responsible. When we name structural racism, we direct attention to the laws, processes, and practices which produce and maintain health inequities (Sewell, 2016, 2015). This offers an incredible opportunity to connect research findings to upstream policies (e.g., non-discriminatory housing laws), thus identifying appropriate points of intervention and moving away from statements related to broad proxies of SEP.

The following recommendations are based upon a diverse array of evidence from previous findings as well as the authors’ beliefs. One highly influential framework is the Public Health Critical Race Praxis model proposed by Ford and Airhihenbuwa (2010a,b). This model states racism is a root cause of social stratification and health inequity and highlights the researcher’s role in either challenging or perpetuating such hierarchies (Ford and Airhihenbuwa, 2010a,b, 2018). If a radical anti-racist framework, such as this model, was applied to neuroscience research then the field could play a larger role in addressing mental health inequities. This will require an unlearning of prior negligent research practices and an ongoing committed effort to learn ethical alternative strategies. While there may be discomfort or defensiveness in interrogating past approaches and holding ourselves accountable in the future, a genuine commitment toward equitable neuroscience research could guide the field forward and further strengthen the interpretative power of studies.

### Report inequities and acknowledge diversity in research samples

In general, few studies examining neighborhood disadvantage have methodologically confronted ethnoracial and socioeconomic inequities (c.f., Harnett, 2020; Taylor et al., 2020; Douglas et al., 2021)—though many call for future work to explore these intersections (e.g., Hunt et al., 2020; Rakesh et al., 2021; Sripada et al., 2021; Webb et al., 2021). Recent theoretical work has proposed moving toward an intersectional neuroscience framework. Such a framework would require reporting and addressing between-group differences in socioeconomic measures in order to help contextualize sample and position inequities at the forefront (Weng et al., 2020). Rooted in Black feminist pedagogy, Crenshaw’s (1991) intersectionality framework was originally used to describe the unique experiences of Black women who experience the intersections of racial and gendered oppression. Within the field of neuroscience, this framework can also be applied to research procedures and methods in order to understand the relationships between systems of oppression related to multiple identities and hierarchies of power and privilege (Carastathis, 2014).

Even outside of the work on socioeconomic factors, reporting of complete demographic variables is not commonplace (Roberts S. O. et al., 2020). Race and ethnicity are still not frequently reported, despite being “required” by many journals. Ethnoracial differences in study measures can only be observed and interpreted if the data are presented. Therefore, we echo calls to report demographic data that is meaningfully and appropriately disaggregated (i.e., based on historical and structural inequities) (Flanagin et al., 2021; Kauh et al., 2021).

The absence of sufficient research on these systemic factors in neurobiology research is also due to the fact that neuroscience research samples are often non-representative of racial and economic diversity within the United States (Henrich et al., 2010; LeWinn et al., 2017; Muthukrishna et al., 2020). This is linked to a history of scientific racism. This history includes the exploitations of communities of color for unethical research purposes and the perpetuation of harmful stereotypes rooted in neuroscience research (Brandt, 1978; Leslie, 1990; Turda, 2010; Saini, 2019). Therefore, improved and intentional recruitment methods are needed to better understand the neural basis of mental health inequities.

Reporting race and ethnicity in neuroscience studies is not enough: proper contextualization of race and ethnicity is essential. In what Nancy Krieger has dubbed “the double-edged sword of data,” structural injustice may operate through data use in one of two ways: (1) preventing documentation that structural injustice exists, and (2) using data in problematic ways that further perpetuate oppression of historically minoritized groups (Krieger, 2021). Undoing these structural issues may be remedied by explaining and justifying the conceptualization and operationalization of racialized groups, and also by analyzing racialized groups in relation to available societal inequity variables (Krieger, 2021).

Specific to neuroscience research, we advocate for more studies to include environmental and structural factors. Critical to this is contextualizing the *racialization* of structural and environmental variables. In the absence of this lens, neuroscience studies attempting to avoid the impact of racism when considering social inequity/disadvantage may reinforce notions of biosocial determinist notions of minoritized groups and being “neurobiologically poor” (Pitts-Taylor, 2019; Krieger, 2021).

### Fund neuroscience work on sociopolitical factors

Support for the inclusion of sociopolitical and structural factors in neuroscience needs to occur not only at the level of specification and analysis, but also at the level of funding and epistemic inclusion. Given that many researchers exploring these topics tend to be members of racialized and historically minoritized groups, the lack of funding to pursue these avenues of research has also been associated with the attrition of diverse scholars (Gilpin and Taffe, 2021). This serves as a disadvantage to the field, as these scholars offer pivotal and unique perspectives that could contribute immensely to the field of neuroscience in general. Greater support from large funding entities will help inform our understanding of the effects of socioeconomic distress on neurobiology across diverse populations.

### Explore resilience factors

Neuroscience research on neighborhood factors has largely focused on risk modeling, evaluating variables believed to worsen mental health. Institutionalized forms of racial inequities, including neighborhood disadvantage, and community violence, are risk factors dominating the emerging field (Butler et al., 2018; Saxbe et al., 2018; Gellci et al., 2019; Wrigglesworth et al., 2019; Borg et al., 2021; Rakesh et al., 2021; Reda et al., 2021; Webb et al., 2021). Discussions backed by **critical race theory** being held in other fields including education, law, and psychology, should inform neuroscience work moving forward (e.g., Yosso, 2005; Gillborn and Ladson-Billings, 2009; Giraldo et al., 2017). A key tenant of critical race theory is that deficit-only perspectives, which minimize the strengths of ethnically and racially minoritized groups/individuals, are harmful (Yosso, 2005; Giraldo et al., 2017). Theoretically, risk-only models are incomplete; and practically, they may further stigmatize marginalized populations. There is ample room and need for resilience modeling (also known as strength-based approach) in studies on socioeconomic circumstances and neurobiology. In the field of neuroscience, exploring the effects of individual, familial, and community factors that are known to mitigate risk of poor mental health outcomes, such as social support/engagement, civic action, critical consciousness, neighborhood cohesion, and racial-ethnic identity, may be extraordinarily beneficial (e.g., Bracey et al., 2004; Dassopoulos and Monnat, 2011; Gapen et al., 2011; Forsyth and Carter, 2012; Johns et al., 2012; Karb et al., 2012; Neblett et al., 2012; Neergheen et al., 2019; Burt et al., 2021; Lardier et al., 2021).

### Engage in community-based participatory and community-engaged research

The final recommendation is the most transformative in the context of traditional Western conceptualizations of research. Human neuroscience has relied primarily on “top-down” scientific processes. In this approach, the power (i.e., decision-making, funding, control over dissemination process, etc.) rests entirely with the study team and its institutions (Wallerstein and Duran, 2010). Although those researched provide data, they are not consulted to ensure the research question(s) or outcomes align with their experiential knowledge or the community’s needs. Even with the best intentions, this Western knowledge production pipeline is inequitable because power is not equally distributed between the researchers and the researched (Minkler and Wallerstein, 2003; Wallerstein and Duran, 2010). Community-based participatory research (CBPR) and Community Engaged Research (CEnR) are different approaches to knowledge production which involve various stakeholders (i.e., community members and academic partners) collaborating throughout the research process (Minkler and Wallerstein, 2003; Wallerstein and Duran, 2010). At its core, CPBR and CEnR hope to build health equity by practicing equity through co-production of knowledge (Minkler and Wallerstein, 2003; Wallerstein and Duran, 2010).

Psychology has started to answer the calls for community-driven research and human neuroscience should follow (Wallerstein and Duran, 2010, 2017; Collins et al., 2018; Arredondo, 2021; Wallerstein, 2021). A first step for research teams is for members to reflect on how their own **positionality** manifests in their work and in interactions with fellow team members and participants (Muhammad et al., 2015). Just as we cannot isolate participants from the sociopolitical environment, we cannot ignore the intrinsic influences of society on research practices or hide behind a façade of self-proclaimed objectivity (Momin, 1972; Muhammad et al., 2015). Furthermore, conducting research without developing proper relationships with the community and necessary scientific experts contributes to “health equity tourism,” which results in diluting existing efforts of committed health equity researchers (Lett et al., 2022). CPBR and CEnR entail community-building (which takes time) as well as sharing wealth and final products (which requires funding and time; Wallerstein and Duran, 2010, 2017; Collins et al., 2018; Wallerstein, 2021) and prioritizing research questions that are important to communities, not researchers.

Within this realm, neuroscience researchers can offer pivotal information on causal mechanisms influencing the neurobiology of disadvantaged groups and further establish the basis for innovative intervention and policy work that can improve the conditions of individuals living amongst socioeconomic distress (Farah, 2018). To make progress in neuroscience community participatory research, funding agencies like the National Institutes of Health must be receptive to funding studies that are likely longer and more expensive. These organizations must also value including community members on research teams, even if these members do not have traditional (i.e., Western knowledge production) research training or traditional indicators of research contributions. As researchers, we can advocate for more funding opportunities while also introducing CBPR and CEnR practices into existing studies (e.g., collaborating with an established community organization during data analysis and dissemination).

## Conclusion

As Angela Davis once noted, “if we are not afraid to adopt a revolutionary stance—if, indeed, we wish to be radical in our quest for change—then we must get to the root of our oppression. After all, radical simply means grasping things at the root” (Davis, 1990). Her call to action—at the time for Black American women—to participate in grassroot organizing, become involved in political/policy work, and serve as activists in order to fundamentally transform socioeconomic conditions contributing to systemic oppression is still very relevant today. We challenge the neuroscience community to also participate in this quest for systemic change. The burden of progressive change is one we all should bear.

The call to address health inequities and build health equity must be met with a radical anti-racist response. As the field of human neuroscience continues to identify biological mechanisms underlying mental health, it must cautiously avoid biological reductionism and essentialism. We encourage all to remain vigilant about discussions of neurobiological effects of sociopolitical variables using only biological terms, and without actually naming oppressive structures (e.g., racism, sexism). In the context of studies on socioeconomic circumstances, defining factors as an institutionalized form of racial inequity (Sewell, 2016) is an initial move toward “grasping at the root” (Davis, 1990). Additional steps include more thorough reporting of demographics which requires comprehensive evaluations of structural and environmental variables. Ultimately, however, more radical anti-racist steps such as challenging Western knowledge production, embracing community research, and reforming funding agencies’ priorities, will lead to transformative change.

## Positionality statement

As in all research, it is helpful to understand the authors’ positionality and, therefore, their lens on the data. All authors are early-career researchers and shared first authorship. EKW is a United States—born white woman, with expertise in investigating associations between sociopolitical factors and neurobiology in the context of mental health inequities. RD is a southern Black American woman, with expertise in community violence, systemic disadvantage, and racial trauma amongst youth of color. CC-I is a first-generation immigrant cisgender man from Mexico who identifies as Mexican and Latinx, and has expertise in neuroscience research exploring structural and environmental factors and their impact on brain development. All authors worked as a team and had regular discussions to ensure the perspective was guided by their collective cultural knowledge and expertise. This was a collaborative team project that ensured the study was sensitive and appropriate to the context in which it was conducted.

## Author contributions

EKW wrote the first draft of the manuscript. All authors wrote the sections of the manuscript, contributed to manuscript revision and approved the submitted version, and shared first authorship.

## Footnotes

1 The majority of work on socioeconomic circumstances and neurobiology has been based in the United States and therefore this paper discusses this research within the American sociopolitical context. However, the authors encourage researchers outside of the United States to consider how global, national, and regional structures of oppression, including racism, may operate, and manifest in research.
